# Need for Improvements in Medical Device Management in Low- and Middle-Income Countries: Applying Learnings from Japan’s Experience

**DOI:** 10.31662/jmaj.2022-0089

**Published:** 2023-03-24

**Authors:** Daisuke Inagaki, Shinji Nakahara, Ung-il Chung, Mikiko Shimaoka, Kuniko Shoji

**Affiliations:** 1 Graduate School of Health Innovation, Kanagawa University of Human Services, Kawasaki, Japan; 2 Center for Research Strategy, Waseda University, Tokyo, Japan

**Keywords:** low- and middle-income countries, healthcare system, technology, innovation

## Abstract

Medical devices provide important infrastructural support in modern healthcare systems. However, in low- and middle-income countries (LMICs), inadequate maintenance and management of devices due to a shortage of healthcare personnel (not only doctors and nurses but also other professionals including biomedical engineers [BMEs]) has resulted in inefficient and weak healthcare systems. High-income countries, including Japan, have resolved these problems by developing human resources and technologies to maintain and manage these systems. In this paper, we discuss the possibility of mitigating these problems in LMICs through human resource development and technology, based on lessons from Japan’s experience. The problem of medical device management in LMICs stems from the fact that there are few professionals, such as BMEs, in charge of the management of medical devices and that clinical engineering departments in charge of device management have not been established. Since the 1980s, Japan has introduced a licensing system for BMEs, establishing operational guidelines to clarify their responsibilities within hospitals and using technology to utilize data and reduce workloads. Nevertheless, workload problems and high costs required to introduce computerized management systems persist. Moreover, it would be difficult to implement the same measures as those adopted in Japan in LMICs where there is an overwhelming shortage of medical personnel. It may be necessary to further reduce workloads for data entry and device management; use up-to-date, inexpensive, and user-friendly technology; and train non-BME personnel to operate and maintain equipment.

## Introduction

Advanced medical devices provide the foundation for modern medicine and need proper maintenance to function effectively ^(1)^. High-income countries (HICs) have responded to this demand by training biomedical engineers (BMEs) in medical device management. However, low- and middle-income countries (LMICs), particularly those with limited resources (i.e., low-income and lower middle-income countries), often face an overwhelming shortage of health personnel, not only doctors and nurses but also BMEs ^(2)^. Consequently, medical devices, (purchased or donated by overseas donors) are often left dysfunctional, unrepaired, or unaccounted for, owing to inadequate maintenance and management. This results in an inefficient and weak healthcare system ^(1), (3)^.

For example, a study in Honduras reported that around 20% of medical equipment was deemed out of service in hospitals without appropriately trained BMEs ^(4)^. A study conducted in Nepal reported that access to spare parts was poor, BMEs had only diploma-level training, and about 14% of equipment was non-functional in hospitals without BMEs ^(5)^. In Uganda, a quarter of BMEs had a bachelor’s degree (the others had a diploma), 86% of the surveyed equipment had no manuals, and 37% of the equipment was non-functional ^(6)^. In Cambodia, where the authors have first-hand experiences, we observed piles of medical equipment in hospital warehouses that were out of order or awaiting maintenance, loss of medical equipment, and the inability to use dialyzers owing to a lack of budget for maintenance.

Although Japan has an adequate medical device maintenance and management system today, it faced similar problems several decades ago: a lack of professionals, difficulties in managing a large number of devices, high costs of medical device management systems, and human error. Japan has gradually solved or at least alleviated these problems by introducing human resource development systems and modern technology, such as the use of databases to manage equipment. In this paper, we describe the problems associated with medical device maintenance and management in LMICs, discuss the history of system improvements in Japan, and examine the possibilities of using the latest technologies to mitigate these problems.

## Device Management Problems in LMICs

International medical cooperation projects provide a range and number of medical devices to LMICs as technology transfer, many of which become dysfunctional before their lifespan ^(1), (3)^. Improper maintenance and incorrect operation of medical equipment have been identified as the main causes of this widespread device malfunction ^(1), (3)^. Without maintenance, parts needing regular replacement are likely to deteriorate. Medical personnel using such equipment often do not learn its proper use, thus causing them to malfunction. Medical equipment donated to LMICs from high-income countries (HICs) is often of various models, second-hand, lacking manuals, and hard to obtain spare parts for, which, in turn, further aggravate the situation.

Proper device maintenance and usage can prevent such malfunction and contribute to reliable clinical practice. Establishing clinical engineering departments in hospitals, designating personnel for medical device management, and making them responsible for periodic inspections, repairs, and education of medical personnel on proper device use help solve this problem. However, in LMICs, BMEs with medical device management expertise and hospitals with clinical engineering departments remain to be extremely scarce. Device management is often outsourced to external organizations ^(1)^. While outsourcing may function as an optimal organizational model depending on the personnel and resource situation, it is difficult to manage all hospital devices or to ensure maintenance quality through outsourcing without an in-house professional who is responsible for the device ^(1)^. Additionally, hospital managers usually allocate low budgets for device management because they do not recognize its importance.

Even in hospitals with clinical engineering departments, engineers/technicians, rather than BMEs, oversee this equipment. Such staff have difficulty maintaining device management quality because of their dearth of medical knowledge and skills to manage medical devices, lack of support from professional associations of BMEs for updating skills and developing their careers, and consequent poor visibility of their roles ^(1), (3)^. Despite efforts to increase BMEs in LMICs, well-trained engineers tend to quit their jobs for better paid positions (a problem common to all medical personnel) ^(2)^.

Insufficient resource input worsens the resource-demand mismatch in device management tasks that are complicated by the large number and range of devices. When a paper-based ledger or simple spreadsheet application is used, all recordkeeping tasks should be done manually, including registering devices and recording information of inspections, maintenance, and utilization, which yields a high workload. In such situations, some devices may easily forego these recording tasks, resulting in skipped regular maintenance or unknown utilization sites.

## Lessons from the History of Japan’s Biomedical Engineer System

In Japan, a licensure system and a standardized educational curriculum to train clinical engineering professionals have been introduced to address this problem ^(7)^. In the 1970s, when sophisticated devices like dialyzers and extracorporeal circulation machines were introduced in clinical practice, BMEs without formal training or licensing began operating and managing these devices. In 1988, the Japanese national licensing system started certifying BMEs as professionals in managing and operating medical devices.

In the United States, the Association for the Advancement of Medical Instrumentation has been certifying clinical engineers since 1970s. Owing to the low reliability of medical devices in the 1970s in the United States, these personnel mainly specialized in the inspection and repair of devices. In Japan, the role of BMEs as technician was different as operating these devices is included in their responsibility.

The Ministry of Health and Welfare issued “Guidelines for the practice of clinical engineering” in 1988 which governed BME practices, although the human resources environments were not sufficiently arranged to properly manage the devices in medical institutions. The Medical Service Act was amended in 2007 to meet the diversified and high demand for medical devices. It required that medical institutions appoint a medical device safety manager responsible for (1) training, (2) formulating and implementing device management plans, and (3) obtaining information and taking measures to improve medical device safety. The revised “Guidelines for the practice of clinical engineering 2010,” specify the methods to manage medical devices through ledgers and record detailed information such as serial numbers, registration numbers, and utilization history.

In terms of ledger management and detailed record entry for a large number and range of devices, BMEs have faced problems associated with manual input and the accompanying correction of numerous errors, which are time consuming. In Japan, recent introduction of computerized management systems has mitigated these issues. These systems enable centralized registration and device management, reducing the time required for device maintenance and input error correction ^(8)^. All device information (e.g., utilization and inspections) can be entered from tablet terminals in the hospital into the system ledger in real time. This allows single data entry even for inspections performed outside the clinical engineering department. Furthermore, creating a database for maintenance and inspection work records of registered devices has made quantitative evaluation of workload and costs possible. Nevertheless, management systems currently in use require manual data input when registering equipment or creating an electronic ledger, which can still result in input errors and a high workload. It also requires a big system implementation budget, which is a major barrier for small- and medium-sized hospitals.

## Recommendations Based on Japan’s Experiences

We recommend developing human resources for medical device management, establishing clinical engineering departments in medical institutions, and disseminating operational support systems (Table 1). The most important aspects of human resource development are the establishment of a long-term plan to train BMEs with a standardized curriculum and the introduction of a licensing system; however, short-term measures are also deemed necessary to address human resource insufficiencies. Few studies show the effectiveness of the deployment or training of BMEs in reducing unfunctional medical equipment. In fact, it was found that BME training programs achieved a 30%‒40% reduction in out-of-service equipment in Rwanda, Honduras, and Cambodia ^(4)^. In Nepal, half as much out-of-service equipment was found in hospitals with BME compared to those without ^(5)^.

**Table 1.** Experience from Japan that Can Be Applied to Alleviate the Problems in Developing Countries.

Problems in developing countries	Solutions in developed countries (Japan’s experience)	Barriers	Future directions
Insufficiently trained human resources in device management	• Standardized training curriculum • Licensing system for biomedical engineers	• Long-term problem of human resource insufficiency • Insufficient training courses for biomedical engineering	• Training of biomedical engineers • Training of non-engineering personnel in device use and management
No personnel or department designated for device management	Compulsory designation of personnel and department in charge of device management in medical institutions	No regulations	Mandate for the establishment of specialized departments and personnel in each facility, and definition of their roles
Arduous tasks in registration and recordkeeping	Use of a computerized management system	High cost of such systems that require a large amount of workload for data input	Inexpensive and user-friendly systems with automated data entry functions

Given the persisting human resource shortage worldwide, we cannot expect only BME personnel to fill these vacancies: it is reported that by 2030, there will be a shortage of 6.1 million healthcare workers in Africa and 4.7 million in Southeast Asia ^(2)^. Therefore, training and retraining of non-BME personnel currently in charge of medical device management should be strengthened. Additionally, medical device utilization education for all medical personnel has been identified to be crucial in LMICs as improper operation of medical devices can lead to their malfunction. However, providing training in terms of the use of hospital devices without BMEs becomes difficult. Since standardized training courses for medical device management are not readily available in LMICs ^(1)^, providing online educational content showing how to use, maintain, and repair medical devices (possibly through smartphones) would be deemed useful. Given the widespread use of smartphones in LMICs, we believe that online educational content accessible from smartphones may prove effective. For instance, a smartphone-based application that provides a step-by-step troubleshooting guide was shown to support BMEs in terms of repairing medical equipment in Ethiopia ^(9)^.

Japan is an example of how human resource development alone is rather insufficient; thus, the creation of supportive environments is deemed crucial ^(7)^. The responsibilities of medical device management should include designating biomedical engineering department and personnel and developing guidelines that clearly define their roles. This is a universal lesson that can be applicable to all countries, although many LMICs have not yet established such supportive environments.

Given the human resource shortage and lack of supportive environments in LMICs, improving operational efficiency is a critical issue. Further, this can possibly be facilitated through the use of new, but inexpensive and accessible technologies. Conventional device management systems, usually based on paper ledgers, have workload problems because of large inputs, which can further increase the burden on those in charge of device management, usually non-BME personnel in many LMICs ^(1)^. This can be overcome with computerized management systems equipped with image recognition technologies using artificial intelligence to improve efficiency. Additionally, computerized system can easily output a list of equipment that is not functioning or requires maintenance. A study in Benin indicated that the introduction of a computerized system has allowed rapid identification and analysis of equipment failure as compared with a paper-based system ^(10)^. For example, a function to recognize and register medical devices from an image of their exterior and legal description label can make the device management system user-friendly by eliminating manual input. Such systems would also be beneficial for small- and medium-sized hospitals even in HICs. However, they should be made inexpensive to ensure that they can be introduced even in the poorest countries.

## Conclusions

In HICs, inadequate maintenance of medical devices in the past has caused malfunction and even harm to patients, but guidelines and legislations have promoted their safe use. Computerized systems have streamlined centralized management of medical devices and information collection. By contrast, insufficient human resources have impeded improvements in device management in LMICs. Advanced technologies from HICs may be deemed useful to solve this problem. However, existing systems still have entry errors, significant input labor, and high costs. Latest technologies like artificial intelligence for image recognition technology may solve such problems and help construct user-friendly systems. We also believe that providing online education regarding medical device management may fill the existing human resource gap in local healthcare systems.

## Article Information

### Conflicts of Interest

None

### Sources of Funding

DI is developing a medical device management system to start up a business supported by the Japan Science and Technology Agency. This work was supported by Japan Science and Technology Agency (the program of Start-up incubation from COre REsearch), Grant Number JPMJST2079.

### Approval by Institutional Review Board (IRB)

Not required because this is an opinion piece.

### Author Contributions

All authors contributed to the conception of this paper, DI drafted the manuscript, and the other authors contributed to the revision of this manuscript. All authors approved the final version.
